# Special Issue: Advances in Transmission Electron Microscopy for the Study of Soft and Hard Matter

**DOI:** 10.3390/ma14071711

**Published:** 2021-03-31

**Authors:** Elvio Carlino

**Affiliations:** Istituto per la Microelettronica ed i Microsistemi, Consiglio Nazionale delle Ricerche (CNR-IMM), Sezione di Lecce, Campus Universitario, via per Monteroni, 73100 Lecce, Italy; elvio.carlino@cnr.it

Transmission Electron Microscopy (TEM) owes its success to the capability to investigate fundamental aspects of nature, answering the human need of knowledge necessary to understand unknown mechanisms and to find new solutions in a variety of fields like physics, biology, medicine, engineering, or chemistry. Since the beginning of modern science, the scientist necessitated to see, in a general sense, the details of a phenomenon to imagine and to develop a model capable of explaining the phenomenon itself. From this point of view, a microscope is the archetype tool capable of studying the ultimate elements of phenomena, which are invisible to the naked eye. When the scientific interest is focused on an atomic scale, this archetypic tool finds its highest expression in the transmission electron microscope. It is worthwhile to remark that the electron microscope itself is nearly useless alone, as it needs microscopy, which is the powerful combination of the most advanced technological equipment for imaging, diffraction, and spectroscopies with the knowledge and the methods necessary to explore all the opportunities provided by the microscope and by the depth of the strong electron-matter interaction. In fact, it is electron microscopy that provides answers to fundamental physical questions, such as the experimental demonstration of the self-interference of the electron [1,2], previously believed possible only as a *gedanken* experiment proposed by Albert Einstein (Richard Feynman is said to have re-marked that self-interference of the electron is the phenomenon that contains everything you need to know about quantum mechanics). Again, it is electron microscopy that provides a vast variety of applications to the study of organic and inorganic samples at an atomic resolution, investigating their shape, crystal structure, chemistry, electric properties, and magnetic properties. From the long story of electron microscopy, it emerges that the conventional use of an electron microscope is a fruitful way to investigate the matter by well-established powerful methodologies, whereas an unconventional use of an electron microscope could sometimes open new routes to new, unexpected knowledge.

This special issue was conceived with this idea in mind, focusing on the advances in transmission electron microscopy, and also scanning transmission electron microscopy (STEM) for the study of both organic and inorganic matter. The purpose is to offer to the scientific community an opportunity to show some of the latest developments in TEM/STEM based methodologies. However, we are conscious that a single issue can cover only a few aspects of this field of investigation. Here, a particular attention is paid to the radiation damage in TEM/STEM experiments, which is currently one of the most limiting factors to the further improvement in spatial resolution and accuracy in atomic resolution imaging and spectroscopy experiments, not only on biological samples.

Within works on approaches to reduce the dose delivered to a specimen, there is a paper [3] dedicated to electron tomography in STEM. Here, electron tomography is applied to seize the global overview of cellular architecture in 3D at the nanometer scale. The need to collect several images with meaningful contrast for 3D tomography is evident in competition with the radiation damage of the sample. Since sparse data collection can perform efficient electron dose reduction, whereas the risk is to lose some information, in Reference [3], the author proposes a method based on compressing sensing or inpainting algorithms for the missing information reconstruction. The method is, hence, applied, as a case study, to a thick biologic specimen.

The paper [4] is an example of how an unconventional use of a standard TEM, equipped with a high coherent electron source, can provide a way to overcome the radiation damage during atomic resolution experiments on single nanoparticles of radiation sensitive, organic, or inorganic matter consisting of low atomic number elements. For these specimens, the nanometric size of the particles and the low scattering power of their constituents jeopardize even the overview of the specimens. In Reference [4], it is demonstrated how in-line holography imaging, performed by conveniently tuning the electron optical conditions, can provide a high contrast overview of the specimen, while delivering a low-density current of electrons of a few e^−^Å^−2^s^−1^. Furthermore, the in-line holograms can be used to tune the electron optical conditions to enable a low dose atomic resolution phase contrast imaging to study the properties of single particles of nano-drugs or of biologic matter.

In-line electron holography, off-axis electron holography, point projection electron microscopy, and electron coherent diffraction imaging are the subjects of paper [5]. In fact, Reference [5] is a review on the theoretical background necessary to understand these approaches and on the recent theoretical and experimental advances in these fields. In view of their importance in the study of radiation-sensitive materials, the significant role of the electron energy has been considered and two ranges of energies of applicative relevance (30–250 eV and 80–300 keV) have been exploited in detail, discussing advantages and disadvantages of the choice of a specific energy, as a function of the specimen of interest. Finally, an explicit comparison between electron holography and electron coherent diffraction imaging has been made both for their capabilities to measure the phase of the electron waves scattered by the specimen and in terms of a minimum dose delivered to the samples.

The paper [6] is an example of application of off-axis holography to the study of magnetic properties of nanoparticles, especially to access and map the magnetic configuration of Fe_3_O_4_ cubic nanoparticles for potential application in magnetic hyperthermia, as a complementary approach to standard therapies for cancer treatment. The advances in the equipment for off-axis holography experiments, providing multiple biprisms for accurate tuning of the field of view and of the experimental setting, enable quantitative mapping of the magnetic properties of single nanoparticles in relationship with the other particles, resulting in the formation of chains, whose shape and size have direct influence for the medical applications. The accuracy of the magnetic mapping makes possible an appropriate comparison with simulations, which is necessary to unveil the complexity of this matter.

One of the reasons for the success of TEM is the possibility to perform several kinds of experiments on the same sample in the same instrument, gaining pieces of cross correlated information, whose ensemble enables us to reach a degree of accuracy and confidence in the knowledge of the properties of a complex specimen, not reachable in a single kind of experiment. The challenging study of the microstructure of amorphous silica was embarked on paper [7] by HRTEM, Electron Energy Loss Spectroscopy and Electron Pair Distribution Function complemented by X-ray powder diffraction.

A single TEM image or spectrum can achieve accurate atomic resolution information on a nanometric volume of the specimen. As a consequence, the experimental strategy of a successful TEM investigation requires us to explore a representative number of regions of interest within the same TEM specimen, and a representative number of TEM specimens, to investigate a general property of the matter under study, and not only a local feature seen by accident on a TEM specimen. Correlative light microscopy and TEM studies are not trivial from an experimental point of view but allow one to complement the peculiarities of two approaches that merge information on the same area achieved with the relevant spatial resolutions. This is what has been investigated in Reference [8], where correlative light microscopy and TEM are successfully applied to the study of selective degradation of mitochondria by autophagy, following the process on a nanometric scale, in cells under stress.

The paper [9] is an example of study on electron energy loss magnetic chiral dichroism (EMCD). EMCD was experimentally demonstrated in 2006 [10], and it is analogous to the X-ray chiral circular dichroism (XMCD), which is an approach, developed about 20 years before, that enabled us to quantitatively study the magnetic phenomena in a correlated electron system by using circularly polarized x-ray photons in a synchrotron. The born of EMCD is another valuable example of how an unconventional use of a TEM can open new ways to understand the nature. The origin of EMCD is related to the observation that the absorption cross section for X-rays and electrons are similar, if we replace the polarization vector for photon absorption with the exchanged momentum in electron impact ionization. This paved the way to discover a method to study the magnetic properties of correlated electrons in solids with a spatial resolution on an atomic scale, which is typical of a TEM. Since the proof of concept of EMCD, the experimental and theoretical advances in this field made EMCD a method currently used all over the world to quantitatively study the magnetic properties of the matter. The paper [9] focuses on the dependence of EMCD on the acceleration voltage and how this basic experimental parameter can be used to optimize EMCD experiments. This is done by deriving an analytic formula for predicting EMCD effects and elucidating the underlying physics, which enables a better tailoring of the electron optical conditions for quantitative EMCD.
